# Dissociating the Neural Correlates of Intra-Item and Inter-Item Working-Memory Binding

**DOI:** 10.1371/journal.pone.0010214

**Published:** 2010-04-19

**Authors:** Carinne Piekema, Mark Rijpkema, Guillén Fernández, Roy P. C. Kessels

**Affiliations:** 1 Donders Institute for Brain, Cognition and Behaviour, Radboud University Nijmegen, Nijmegen, The Netherlands; 2 Department of Experimental Psychology, Oxford University, Oxford, United Kingdom; 3 Department of Neurology, Radboud University Nijmegen Medical Centre, Nijmegen, The Netherlands; 4 Departments of Medical Psychology and Geriatric Medicine, Radboud University Nijmegen Medical Centre, Nijmegen, The Netherlands; University of Groningen, Netherlands

## Abstract

**Background:**

Integration of information streams into a unitary representation is an important task of our cognitive system. Within working memory, the medial temporal lobe (MTL) has been conceptually linked to the maintenance of bound representations. In a previous fMRI study, we have shown that the MTL is indeed more active during working-memory maintenance of spatial associations as compared to non-spatial associations or single items. There are two explanations for this result, the mere presence of the spatial component activates the MTL, or the MTL is recruited to bind associations between neurally non-overlapping representations.

**Methodology/Principal Findings:**

The current fMRI study investigates this issue further by directly comparing intrinsic intra-item binding (object/colour), extrinsic intra-item binding (object/location), and inter-item binding (object/object). The three binding conditions resulted in differential activation of brain regions. Specifically, we show that the MTL is important for establishing extrinsic intra-item associations and inter-item associations, in line with the notion that binding of information processed in different brain regions depends on the MTL.

**Conclusions/Significance:**

Our findings indicate that different forms of working-memory binding rely on specific neural structures. In addition, these results extend previous reports indicating that the MTL is implicated in working-memory maintenance, challenging the classic distinction between short-term and long-term memory systems.

## Introduction

An important quality of human cognition is the ability to associate and integrate different aspects of an experience into one coherent episode. The separate features of items, such as spatial characteristics, shape, colour, and semantic content, are generally processed in specialized regions distributed across the brain [1], and have to be bound together as conjunctions in order to be perceived and stored as part of a coherent episode [2]. Furthermore, the binding of different items - i.e., forming associations or relations between items–is also crucial for the formation of episodic memories.

The MTL has been implicated in binding operations within long-term memory [3]–[5], but evidence on the specific nature of its contribution to binding operations in working memory has emerged only recently. That is, lesion data have demonstrated deficits in short-term maintenance of conjunctions in patients with hippocampal amnesia [6] and a neuroimaging study comparing young and older adults revealed differences in hippocampal activation during working-memory binding [7]. However, most studies only examined spatial binding, although it has been suggested that different binding mechanisms may exist that rely on distinct brain regions [8]. Indeed, in a previous working-memory study, we showed that the MTL was more active in a condition that required the association of a number and its location on the screen (spatial binding) as compared to associations formed between a number and its colour (non-spatial binding) [9].

This pattern of results has two possible interpretations. The first highlights the specific role of the hippocampus in spatial processing [10], [11], that is, the activation of the MTL is driven by the spatial nature of the binding. The second is based on the notion that different forms of memory binding exist, which may differentially rely on hippocampal activation [8]. Different distinctions can be made here. For example, a distinction can be made between intra- and inter-item associations [12]. Intra-item associations are associations between objects and their features that, when bound together, are commonly perceived as a single entity [13]–[16], often referred to as ‘object tokens’ [8]. With respect to intra-item binding, a further distinction can be made. That is, object features can either be intrinsic or extrinsic. Intrinsic features are an aspect of the item itself (e.g., the colour), extrinsic features are strictly speaking not part of the item, but are defined by the spatiotemporal characteristics of the event [17], [18]. While there is no consensus with respect to the precise definition of intrinsic versus extrinsic features (see, e.g., [18], for an extensive discussion), it can be argued that intrinsic features modify the content and meaning of the item directly, whilst extrinsic features do not. In turn, inter-item associations are relational configurations between separate items [19]–[21] that constitute an episode and can be regarded as ‘episodic tokens’ [8].

More recently, Mayes et al. [5] distinguished two different types of associations with distinct characteristics, and possibly different neural correlates. According to this view, associations between items can either be within- or between-domain. Within-domain associations are formed between the same or similar types of items or features (e.g., face/face associations) that are likely to be represented by activity in the same or closely interacting neocortical structures. Between-domain associations are formed between different types of items or features that are represented in distinct neural modules (e.g., face/house associations) and normally processed in anatomically separate structures [5]. Since the items that constitute a between-domain association are processed in different regions across the brain, a distinct neural structure (the MTL) may be required for their integration [5]. Alternatively, within-domain associations describe a type of relationship between items that share an overlapping neural representation (e.g., two objects from the same semantic category) that does not require a distinct “binding module” for their integration.

**Figure 1:**
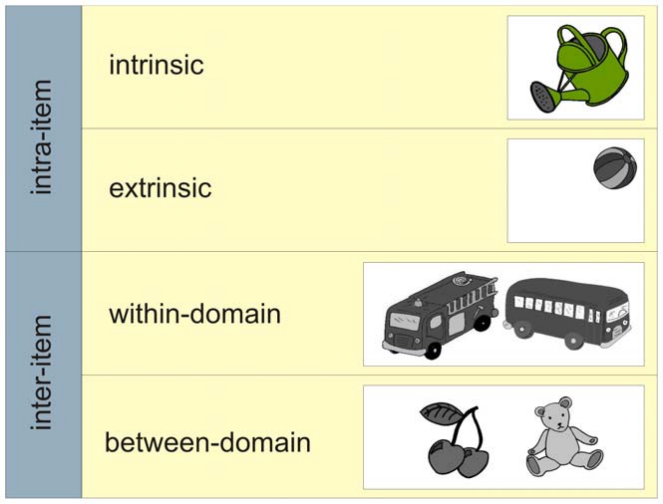
Taxonomy of types of relational memory, distinguishing intra-item binding (objects and their features that can be either intrinsic or extrinsic) and inter-item binding (two unrelated items that have highly similar characteristics–within-domain–or have less overlap with respect to stimulus characteristics–between-domain).

Figure 1 shows a schematic taxonomy of these different forms of binding. While it can be argued that different levels of binding exist at a conceptual level [8], it is still an open question whether these different forms of binding can be dissociated at a neural level as part of working-memory maintenance. For instance, the binding of objects and their locations can be seen as a form of intra-item binding in which and item (the object) is bound with an extrinsic feature (its location). However, the processing of object information and spatial information occurs in different modules within the brain. Consequently, it may be that the formation of intra-item object-location conjunctions relies on the MTL binding module to the same extent as the formation of inter-item between-domain associations (e.g., between a face and a name). To date, however, there are no studies that directly compare these different forms of binding.

In the present study, we used a delayed-match-to-sample (DMS) task, comparing an intrinsic intra-item binding condition (object/colour), an extrinsic intra-item binding condition (object/location), and a between-domain inter-item binding condition (object/object), with a non-binding condition as baseline. We focused on the delay period of a working memory task to avoid neural activity related to stimulus perception or long-term memory processing as much as possible. Since the MTL is particularly important for spatial associations, one can on the one hand expect this region to be activated more in the extrinsic intra-item binding condition (object/location) than in the intrinsic intra-item (object/colour) and the between-domain inter-item (object/object) conditions. If on the other hand the integration of representations does not overlap as a result of feature characteristics that crucially recruit the MTL, one would expect that both the extrinsic intra-item binding and the between-domain inter-item conditions activate the MTL. In line with our previous study [9], we do not expect involvement of regions within the medial temporal lobe to mediate the binding of intrinsic intra-item features.

## Methods

### Ethics statement

All participants provided written informed consent in accordance with the declaration of Helsinki. This study was approved by the local ethics committee (CMO region Arnhem-Nijmegen, Netherlands).

### Participants

Eighteen right-handed healthy university students (4 males, age-range 18–28 years, mean age 23 years, 1–5 years of university level education) without neurological or psychiatric history (self-report) and normal or corrected-to-normal vision participated in the experiment.

### Experimental setup

During scanning, participants lay comfortably in a supine position in the MR scanner. An adjusted padded head holder restricted head movement. Visual stimuli were projected onto a translucent screen at the back of the scanner and seen by the participants through a mirror. All stimuli were presented on a black background. Button press responses were recorded via an MR compatible keypad.

**Figure 2:**
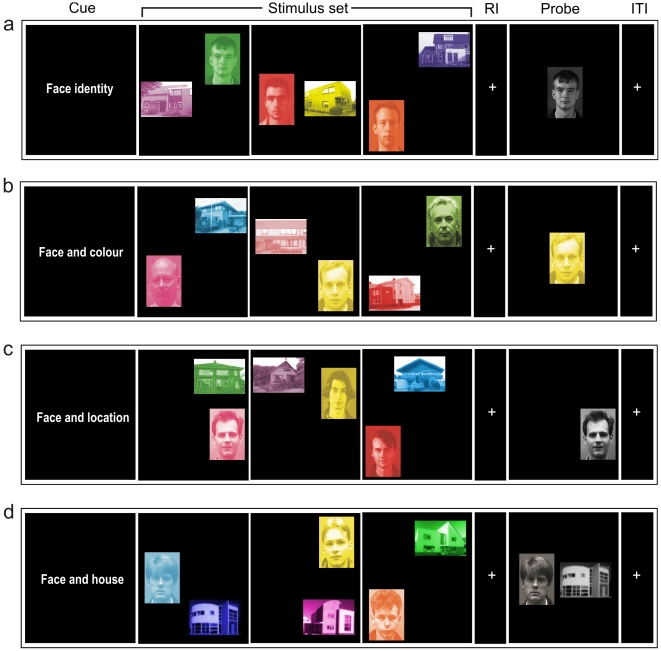
Graphical depiction of the experimental procedure (3-item DMS task). Each of the 3 items was composed of a face and a house, which were presented in one of eight colours (random) and one of eight locations on the screen (random; 2 s ‘item’ stimulus set duration 6 s). A cue (1 s) at the start of each trial indicated which feature or combination of features of the stimulus set had to be maintained. After the retention interval (RI, 10 s) the probe (2 s) came on the screen and the participant had to indicate by button press (right hand only) whether the current stimulus set included the particular item or combination of items. Trials were separated by an inter-trial interval (ITI; jittered between 3–5 s). An example of a match trial is given for each type of condition: a) single item non-binding (baseline), b) intrinsic intra-item binding, c) extrinsic intra-item binding, and d) inter-item binding. The photographs of the faces depicted were taken from the Face Recognition Database available at www.face-rec.org/databases.

Participants performed a 3-item DMS task [22] with faces and houses as stimulus material, henceforth referred to as objects. The task consisted of a single item non-binding condition (face/house-identity) and three binding conditions (i.e. object/colour, object/location, and object/object binding) (see Figure 2 for a schematic overview). Stimuli were randomly selected from a set of eight different photos of houses (visual angle∼6°) and eight different photos of faces (∼6°), each dyed separately in eight different colours (green, yellow, orange, red, pink, purple, blue, cyan) and presented at eight locations on the screen. The face photographs showed a close-up of a male adult face and part of the neck, and the house photographs were close-ups of modern day homes. Stimuli were presented at the corners of a horizontally and vertically centred, symmetric, invisible octogram (∼20° off centre). All stimuli presented in one trial never had the same colour, meaning that within one screen the face and the house had different colours too. We used a design with a factor Association with three levels, and the single-item condition (i.e., the participant had to maintain the identity of the presented faces or houses) as baseline. Thus, the experiment included four conditions. The participants were instructed to retain (1) the identity of the face or the house presented (baseline), (2) the combination of the identity of the stimuli and their colour (object/colour: intrinsic intra-item binding), (3) the combination of the identity of the stimuli and their location (object/location: extrinsic intra-item binding), and (4) the combination of the identity of the face and the house presented together (object/object: inter-item binding).

A cue presented for one second at the start of each trial indicated which stimulus feature or combination of features had to be retained. When a combination of an object with either colour or location had to be maintained, it was indicated whether the subject had to maintain those features for the faces or for the houses. Thus, of each of the three screens presented in one trial the subject was required to only encode and maintain two features. Subsequently, three pairs consisting of a face and a house were presented sequentially (2 s per display). Following the delay (10 s), during which the participant was fixating on a centrally presented cross, the probe was presented (2 s). This was either a gray-scaled face or house presented in the middle of the screen (baseline condition), a coloured object presented in the middle of the screen, a gray-scaled face and a gray-scaled house presented in the middle of the screen side-by-side, or a gray-scaled object (either a face or a house) presented at a certain location on the screen. Participants were instructed to indicate by button press whether the feature or specific combination of features as specified by the initial cue belonged to the current stimulus set, or not. If the probe did not match a stimulus from the set, it was composed of a recombination of the features/stimuli presented in the set. The inter-trial interval (ITI) varied between 3 and 5 seconds (uniform distribution).

This setup allowed us to distinguish sustained blood oxygen level dependent (BOLD) responses associated with the delay interval from transient responses associated with the memory set and the probe stimuli [23]. During scanning, 136 trials from the four conditions (34 trials per condition, 50% matches) were presented randomly intermixed. A unique stimulus list was created for each participant, randomizing objects, colours, locations and ITI lengths.

To familiarize the items beforehand, the participants were presented with a sheet of all possible faces and all possible houses prior to the start of the experiment. To acquire the task instructions, a training session (different set) was performed outside the scanner. Participants were instructed to prioritize correct responding over speed.

### fMRI Data Acquisition

During MRI scanning whole head T2*-weighted EPI-BOLD fMRI data were acquired with a Siemens Sonata 1.5 T MR-scanner (Siemens, Erlangen, Germany; 33 axial slices, ascending acquisition sequence, TR = 2.29 s, TE = 30 ms, 90 degree flip-angle, slice-matrix size = 64×64, slice thickness = 3.0 mm, slice gap = 0.5 mm, field of view = 224 mm). Following the experimental session, a high-resolution structural scan was acquired, using a T1-weighted MP-RAGE sequence (TR = 2250 ms, TE = 3.93 ms, 15 degree flip-angle, 176 sagittal slices, slice-matrix size = 256×256, slice thickness = 1 mm, slice gap 0.5 mm, field of view = 256 mm).

### Data Analysis

For the behavioural data analysis, error rates per condition, acquired during scanning, were subjected to a repeated measures analysis of variance with the factor Association. Significant effects were investigated using paired-sample *t*-tests.

With respect to the fMRI analyses, image pre-processing and statistical analysis were performed using the SPM5 software (www.fil.ion.ucl.ac.uk/spm). The first five volumes of each participant's functional data were discarded to allow for T1 equilibration. Preprocessing consisted of the following steps: realignment, slice-time correction, co-registration of the structural MR image to the mean of the functional images, spatial normalization into MNI space, and spatial smoothing with an 8 mm FWHM kernel of the functional data.

fMRI data were analyzed using the general linear model [24]. We created a design matrix in which for each of the four conditions we modelled the cue-period (consisting of the cue and the stimulus set), the delay period, the probe period (including the button press for the response), and the inter-stimulus-interval separately for correct and incorrect trials, allowing us to look at the different stages of a trial for each condition separately. In addition, the realignment parameters were included in the model to account for movement-related variability. A high-pass filter (128 s) was applied to account for low-frequency effects.

Contrast images of delay-related effects of each of the three conditions with associations (correct trials only) were created, and entered into a second-level analysis, treating subjects as a random variable. Hence, we are probing brain activity related to active and sustained maintenance of different types of associations in working memory. Non-sphericity correction for correlated repeated measures was applied. Unless otherwise specified, we used both the cluster-size and local maximum as test statistics. All *p*-values are corrected for multiple non-independent comparisons based on the theory of smooth random fields (*p* = 0.05 FDR-corrected) [25].

## Results

### Behavioural data

Consistent with earlier studies using a similar task, participants performed significantly above chance level (50%) in all conditions [(1) baseline (single object): mean correct 93.5% (SD 4.4%), *t*(17) = 42.0, *p*<0.0001, (2) object/colour: mean correct 79.9% (SD 5.9%), *t*(17) = 21.3, *p*<0.0001 (3) object/location: mean correct 88.8% (SD 6.4%), *t*(17) = 25.7, *p*<0.0001 and (4) object/object: mean correct 76.2% (SD 8.9%), *t*(17) = 12.6, *p*<0.0001]. The conditions in which two items had to be bound together were significantly more difficult than the single-item condition (*t*(17) = 4.0, *p* = 0.001). Comparisons between the three multiple conditions revealed that recognition performance varied significantly between object/location and object/colour binding (*t*(17) = 5.4, *p*<0.0001) and between object/location and object/object binding (*t*(17) = 6.0, *p*<0.0001), but only marginally between object/colour and the object/object binding (*t*(17) = 1.9, *p* = 0.071).

### fMRI Data

Whole brain analysis showed that a variety of regions commonly associated with working memory maintenance (for review see [26]) were active when all delay periods on correct trials of associative working memory were compared to the baseline, indicating that our set-up was suitable to assess the brain regions involved in working memory maintenance. The local maxima of the activated cluster for this analysis are listed in Table 1 and are corrected for multiple comparisons (FDR = 0.05). Since we were interested in the active maintenance the focus for the analysis was on the delay period. To reveal neural activity related to each binding condition, the contrasts on the second level were created in such a way that one association type was contrasted against the other two, resulting in three different contrasts, namely, *intrinsic intra-item binding* (object/colour vs. object/object + object/location), *extrinsic intra-item binding* (object/location vs. object/colour + object/object), and *inter-item binding* (object/object vs. object/colour + object/location).

**Table 1:** General activation related to the delay period collapsed over all trials against a low level visual fixation baseline.

Anatomical region	MNI Coordinates			T value	Number of voxels
	x	y	z		
Left superior parietal lobule (BA7)	−18	−72	56	6.46	1454
Precuneus (BA7)	12	−72	52	6.16	
Right superior parietal lobule (BA7)	20	−64	54	6.00	
Left middle frontal gyrus (BA6)	−24	−2	58	5.67	170
Cuneus (BA17)	8	−98	8	5.35	61
Left middle temporal gyrus	−32	−74	26	4.18	106
Left superior frontal gyrus (BA6)	−4	14	60	5.17	59
Left inferior frontal gyrus (BA9)	−36	4	30	5.16	106
Left inferior parietal lobule (BA40)	−46	−36	46	4.97	183
Right middle occipital gyrus (BA19)	28	−88	18	4.48	43
Right fusiform gyrus (BA37)	54	−62	−14	4.45	5
Right superior frontal gyrus (BA8)	28	4	50	4.43	33
Right lingual gyrus (BA8)	16	−86	4	4.40	22
Right insula (BA13)	32	22	12	4.39	44
Left fusiform gyrus (BA36)	−44	−40	−20	4.24	8

All reported activations are whole brain corrected for multiple comparisons (FDR<0.05).

**Figure 3:**
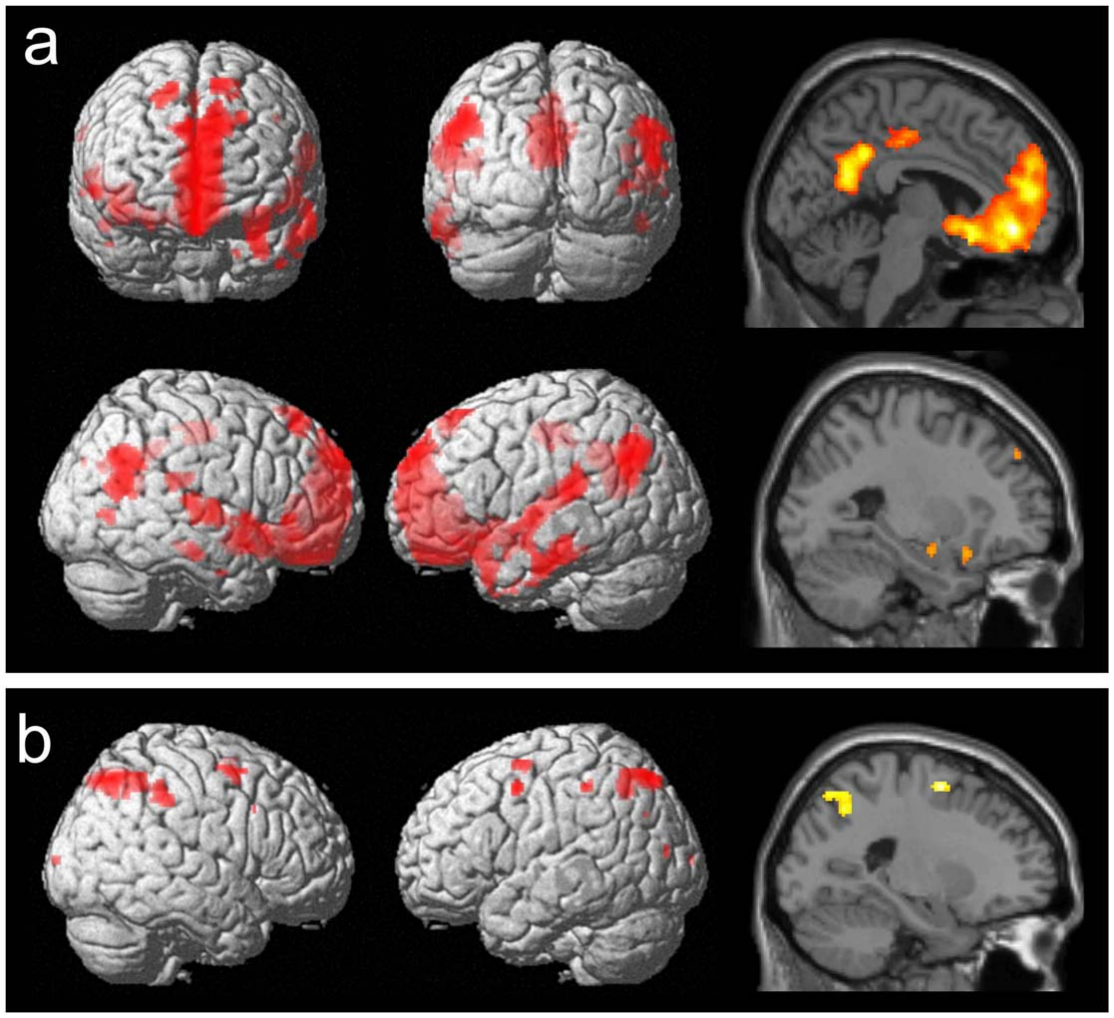
a) the activation related to the inter-item binding contrast rendered onto the surface of the canonical brain provided by SPM, and selected slices depicting the extended midline and medial temporal lobe activation (x = −4; x = 28); b) the activation related to the extrinsic intra-item binding contrast rendered onto the surface of the canonical brain and on a selected sagittal slice (x = −22).

As shown in Figure 3 and listed in Tables 2 and 3 the contrast inter-item binding revealed a large pattern of activation within the prefrontal cortex, the ventromedial prefrontal cortex, the medial temporal lobe, and lateral temporal regions extending into the angular gyrus. The contrast extrinsic intra-item binding specifically activated the superior parietal lobe extending into the precuneus, and the frontal eye fields bilaterally. The contrast intrinsic intra-item binding did not reveal any significant activation, even at a very lenient threshold of *p*<0.1.

**Table 2:** Differential activity related to the contrast extrinsic intra-item binding (object/location vs. object/colour + object/object).

Anatomical region	MNI Coordinates			T value	Number of voxels
	x	y	z		
Superior parietal lobe/Precuneus*	−12	−76	50	4.74	701
	14	−72	52	4.72	
	18	−64	54	4.73	
Frontal eye fields*	−22	0	60	4.89	54
Frontal eye fields (right)**	30	4	56	4.40	46

*Corrected for multiple comparisons at the cluster level.

**p<.001 uncorrected.

All others FDR<0.05 corrected for multiple comparisons.

**Table 3:** Differential activity related to the contrast inter-item binding (object/object vs. object/colour + object/location).

Anatomical region	MNI Coordinates			T Value	Number of voxels
	x	y	z		
Superior frontal gyrus (BA8)	14	39	45	4.78	84
	−10	35	45	4.93	95
	42	33	−10	3.93	48
	−8	57	21	6.64	
Medial frontal gyrus (BA11)	−4	42	−11	7.10	5127
	0	38	−19	6.72	
Inferior frontal	−34	19	−12	5.14	263
	57	33	−2	3.95	31
Precentral gyrus (BA4)	50	−22	−14	4.69	42
Posterior Cingulate	−6	−53	21	5.88	1404
	−4	−43	31	5.49	
	−2	−17	40	5.19	
Superior temporal gyrus/Angular gyrus	−48	−64	34	5.76	528
	−40	−57	31	5.71	
Middle occipital gyrus (BA37)	42	−68	5	3.83	27
Inferior temporal gurus/Superior temporal gyrus/Fusiform gyrus	−51	−8	−1	6.24	475
	−53	−17	−23	5.13	
	−36	−24	−14	5.56	
Inferior temporal gyrus	57	6	0	3.39	12
Superior temporal gyrus (BA41)	51	−23	8	4.45	357
	57	−9	6	3.68	
	44	−13	2	3.66	
Superior temporal gyrus (BA38)	−36	3	−17	3.40	12
Supramarginal gyrus (BA40)	57	−51	29	3.93	374
	51	−59	32	4.92	
	57	−57	20	4.86	
Superior temporal gyrus (BA42)	−57	−27	13	4.62	519
Middle temporal gyrus (BA21)	−48	10	−36	3.69	10
Amygdala	−26	−6	−11	3.69	25
Parahippocampal gyrus (BA36)	28	−28	−12	3.73	17

All activations are corrected for multiple comparisons (FDR<0.05).

## Discussion

In the current study we set out to investigate the involvement of the MTL in binding in working memory by contrasting directly whether the hippocampal activation depends on the presence of a spatial component, or that the MTL is implicated in the binding non-overlapping neural representations in general. We hypothesized, based on previous findings discussed in the introduction, that if the spatial component is a necessary prerequisite for the involvement of the MTL in the process of binding in a working memory task, the extrinsic intra-item (i.e., spatial) binding condition would activate the MTL more when compared to the activation in the other conditions. However, when the MTL is important for the binding of non-overlapping neural representations, one would expect the MTL to be activated during the maintenance of both the extrinsic intra-item and the inter-item binding condition.

Contrasting the conditions against each other revealed very different patterns of activation for each condition. In the inter-item binding condition we observed activation in the MTL, including the parahippocampal gyrus and the amygdala, suggesting that the process of binding of two unrelated objects, a face and a house, depends on the MTL. Other activations for this condition were observed in the prefrontal cortex, the ventromedial prefrontal regions, superior parietal cortex, and lateral temporal regions extending into the angular gyrus. This pattern of activation has previously been observed in associative long-term memory [27], [28], and working memory [29]. The ventromedial prefrontal regions have recently been implicated in self-projection, the ability to remember the past, think about the future, and imagine the viewpoint of others [28], [30], [31]. These processes require a flexible memory system with which we are able to use previous knowledge for future experience. In the current study a small number of items or features form a large number of associations by permutations and thus participants have to ‘forget’ associations learned in previous trials in order to perform well on the current one. Thus, we suggest that the pattern of activation observed in this condition is also related to the flexibility required by this particular task.

Areas implicated in spatial processing, including the superior parietal lobe extending into the precuneus, and the frontal eye fields (FEF) were activated in the extrinstic intra-item binding condition (object/location). However, no activation was observed in the MTL in this condition. The discrepancy between the results of the current and our previous study [9] may be related to the differences in the duration of the delay periods used in both studies. In the current study a fixed delay of 10 seconds was used, whereas in the previous study a jittered delay lasted from 8 up to 20 seconds. Such an unpredictable and on average long delay might particularly engage the MTL [32]. The superior parietal lobe has been implicated in directing visual attention to locations [33], and activation in this region often co-occurs with activation in the dorsolateral frontal regions (i.e., FEF) [34]. Numerous event-related fMRI studies have provided consistent evidence that neuronal activation in the FEF persists throughout the entire delay period of a spatial working memory task, and it is thought to represent the maintenance of the spatial information in motor or saccadic coordinates [35]. Thus, this network may support the maintenance of the location of an item in a working memory task by sustaining covert attention to a particular location [36].

In compliance with the results of our previous study, no specific neural correlates involved in the maintenance of the intrinsic intra-item binding condition were observed. While the interpretation of null results should always be done with caution and these findings do not necessarily indicate the absence of an effect, we suggest that the colour and the object may already be integrated in higher-order visual processing stages [37], [38], forming a unitized object and may thus be processed as a single item [5], which may not require additional processing further downstream.

In all, our results clearly show that different binding processes in working memory evoke different patterns of activation in brain activation. Although one would expect to find MTL involvement in the binding of neurally non-overlapping representation in general, we suggest that it is more likely that the MTL is involved in the binding of inter-item associations compared to extrinsic intra-item binding alone. It should be noted that performance differences were found between the various task conditions, with the most difficult task condition showing the highest activation pattern. Differences in performance may potentially affect activation maps though contamination by erroneous responses [39], making the interpretation of findings difficult. However, in all contrast analyses we only included correct trials to overcome this potential confound. In addition, our findings are in agreement with other studies highlighting the role of the hippocampus in working-memory processing. Working-memory impairments for complex visual stimuli have been demonstrated in patients with MTL damage [40], [41], as well as deficits in short-term maintenance of conjunctions in MTL amnesia [40] and Alzheimer-type dementia which is characterized by MTL atrophy [42]. Furthermore, Nichols et al. [43] showed that working-memory maintenance and episodic-memory formation relied on the same hippocampal region.

However, the interaction between working memory and episodic memory is still poorly understood. In an attempt to overcome this theoretical gap, Baddeley [44] has added a new component, the episodic buffer, to his working-memory model. This buffer is a processing component that is involved in the integration of information from multiple domains into a unified representation that eventually is stored as an episode in long-term memory. In this view, the episodic buffer may act as an intermediate store between short-term maintenance and long-term storage. From a computational perspective, it has been previously proposed that the hippocampus may act as the buffer between working-memory and episodic memory [45], resembling Baddeley's concept of the episodic buffer (but see [46]). Although this notion is in agreement with the suggestion that working memory is nothing more than activated long-term memory [47], the role of the hippocampus in this respect is unclear [48] and empirical studies are lacking. Moreover, one can argue that the MTL does not support specific working-memory processes, but that long-term encoding occurs during delayed maintenance tasks. While the classic dissociation between working memory and long-term memory is currently under debate [49], future studies on working-memory binding in relation to episodic-memory formation may shed further light on the underlying neurocognitive mechanisms of the MTL memory circuit.
